# Revised Atomic Charges for OPLS Force Field Model of Poly(Ethylene Oxide): Benchmarks and Applications in Polymer Electrolyte

**DOI:** 10.3390/polym13071131

**Published:** 2021-04-02

**Authors:** Chan-En Fang, Yi-Chen Tsai, Christoph Scheurer, Chi-Cheng Chiu

**Affiliations:** 1 Department of Chemical Engineering, National Cheng Kung University, Tainan 70101, Taiwan; samuel083826@gmail.com (C.-E.F.); a4775324@gmail.com (Y.-C.T.); 2 Hierarchical Green-Energy Materials (Hi-GEM) Research Center, National Cheng Kung University, Tainan 70101, Taiwan; 3 Chair for Theoretical Chemistry and Catalysis Research Center, Technische Universität München, Lichtenbergstrasse 4, D-85747 Garching, Germany; scheurer@fhi.mpg.de

**Keywords:** polyethylene oxide, OPLS force field, dielectric constant, molecular dynamics simulations, polymer electrolyte

## Abstract

Poly(ethylene oxide) (PEO)-based polymers are common hosts in solid polymer electrolytes (SPEs) for high-power energy devices. Molecular simulations have provided valuable molecular insights into structures and ion transport mechanisms of PEO-based SPEs. The calculation of thermodynamic and kinetic properties rely crucially on the dependability of the molecular force fields describing inter- and intra-molecular interactions with the target system. In this work, we reparametrized atomic partial charges for the widely applied optimized potentials for liquid simulations (OPLS) force field of PEO. The revised OPLS force field, OPLS^R^, improves the calculations of density, thermal expansion coefficient, and the phase transition of the PEO system. In particular, OPLS^R^ greatly enhances the accuracy of the calculated dielectric constant of PEO, which is critical for simulating polymer electrolytes. The reparameterization method was further applied to SPE system of PEO/LiTFSI with O:Li ratio of 16:1. Based on the reparametrized partial charges, we applied separate charge-scaling factors for PEO and Li salts. The charge-rescaled OPLS^R^ model significantly improves the resulting kinetics of Li^+^ transport while maintaining the accurate description of coordination structures within PEO-based SPE. The proposed OPLS^R^ force field can benefit the future simulation studies of SPE systems.

## 1. Introduction

Solid polymer electrolytes (SPEs) have attracted wide attention due to their high safety, low leakage issue and good mechanical strength compared with conventional liquid electrolytes [1,2]. SPEs have been applied in developing various energies devices such as lithium-ion batteries, solar cells, and supercapacitors [3,4]. Among various SPEs, poly(ethylene oxide) PEO, or poly(ethylene glycol) PEG, is one of the most common polymer hosts for its low toxicity, good electrochemical stability, and the excellent ability to dissolve various lithium salts [5,6]. PEO can form stable complexes with Li^+^ via its ether oxygen atoms and conduct ions along its flexible chains [7,8]. Poly(ethylene oxide) dimethyl ether, PEODME, denotes the aprotic PEO methylated at both ends. Short chained PEODME, also referred as glyme, has less interaction with anions due to lack of terminal hydroxyl groups [9,10]. Glyme has been commonly applied in room temperature ionic liquids or as plasticizers for SPE to enhance the ion conductivity [11,12,13,14]. Please note that at high molecular weight, PEODME and PEO show similar physico-chemical characteristics as polymer electrolyte for the minimal effects of terminal groups in long-chain polymeric systems [15,16,17].

Molecular dynamics (MD) simulations can provide microscopic insight into polymer electrolyte systems and have contributed to unveiling the molecular mechanisms of ion transport within SPEs [7,18,19]. To better understand the thermodynamic and dynamic properties of target systems, the reliability of force field potentials describing inter- and intra-molecular interaction is critical. The all-atom optimized potentials for liquid simulations (OPLS) force field is one of the most common molecular models applied in MD studies of polymers, organic solvents, ionic liquids, and SPE systems [20,21,22,23,24,25]. Acevedo et al. developed OPLS models for various ionic liquid molecules that can accurately reproduce experimental properties, including density, heats of vaporization, heat capacities, and viscosities, etc. [20,23]. For the PEO system, various modifications have been proposed to enhance the applicability of OPLS model for electrolyte systems. Saito et al. revised the van der Waals parameters and atomic partial charges for OPLS to study the Li^+^ complex structure with glyme in ionic liquid and demonstrated consistent results with experiments [12]. Tsuzuki et al. modified OPLS for short-chain PEOGME, triglyme (G3), and tetraglyme (G4), to examine the coordination structures and ion dynamics within [Li(glyme)]^+^TFSI^−^ (bis(trifluoromethane)sulfonimide) ionic liquid [13]. Note, however, a later report by Barbosa et al. showed that the original OPLS force field is more accurate in calculating density, thermal expansivity, and dielectric constant of pure glyme systems than the two modified OPLS models [26].

Polarizable force fields allow atomic charge variations and have been successful in modeling various PEO-based SPE systems, including PEO/LiPF_6_, PEO/LiTFSI, and PEO/LiBF_4_, etc. [27,28,29,30]. In contrast, non-polarizable force fields, including OPLS, assign full charges for ions and fix the partial charge of each corresponding atom. Compared with polarizable force fields, non-polarizable models are more computationally efficient. Yet, the dynamics from MD simulations with non-polarizable force field have been shown to deviate substantially from experimental data [31,32]. To overcome this problem, a common strategy is to rescale the atomic charges within the system by a factor of 0.5–0.8 to account for the charge transfer among ion pairs [23,24,33,34]. However, reducing charges may correct the dynamics but also induce improper description of system structural properties [35,36]. Therefore, choosing appropriate scaling factors is crucial for simulation studies of electrolyte systems using non-polarizable force fields.

As discussed above, deriving atomic partial charges is critical in force field development, particularly for electrolyte systems in which electrostatic potentials play major roles in inter-molecular interactions. A recent study by Barbosa et al. developed transferable parameters of short-chain PEOGMEs based on the general AMBER force field (GAFF), another common force field for organic compounds, and derived the atomic partial charges via the restrained electrostatic potential (RESP) method [26]. The modified GAFF for various glymes showed significant improvement over the original GAFF in the calculations of density, viscosity, vaporization enthalpy, and dielectric constant. Another study by Liu et al. reparametrized OPLS force field for chlorinated hydrocarbons with RESP partial charges [37]. The revised OPLS force field greatly improved the accuracy on reproducing experimental dielectric constant values. Inspired by these works, here we revised OPLS parameters of long-chain PEODME using RESP partial charges. The newly derived model, i.e., OPLS^R^, was tested for its predictability of density, phase transition, thermal expansion coefficient, and dielectric constant of the pure PEO polymer melt system. The reparameterization was further applied to the PEO/LiTFSI SPE system with O:Li ratio of 16:1. Based on the resulting RESP partial charges, we propose to use different charge-scaling factors for PEO and Li salts. The charge-rescaled OPLS^R^ model for SPE significantly improves the prediction of Li^+^ dynamics without sacrificing the microscopic description of PEO-based SPE structures.

## 2. Methods

### 2.1. Force Field and Atomic Charge Reparameterization

In the non-polarizable OPLS force field, the system energy is calculated as the sum of all intra-molecular and inter-molecular interactions [20,23,38,39]. The intra-molecular terms, $E_{bond}$, includes bond stretching, angle bending, and torsional energies. The inter-molecular potential, $E_{nonbond}$, includes Coulomb energies and van der Waals interaction described by 12-6 Lennard-Jones terms:

(1) $$E_{sys}=E_{bond}+E_{nonbond},$$

(2) $$\begin{array}{cc} E_{bond}= & \sum_{i}k_{b,i}{\left(d_{i}-d_{0,i}\right)}^{2}+\sum_{j}k_{a,j}{\left(\theta_{j}-\theta_{0,j}\right)}^{2}+\\ \; & \sum_{m}\frac{1}{2}\left[k_{1,m}\left(1+cos\phi \right)+k_{2,m}\left(1-cos2\phi \right)+k_{3,m}\left(1+cos3\phi \right)+k_{4,m}\left(1-cos4\phi \right)\right], \end{array}$$

(3) $$E_{nonbond}=\sum_{i}\sum_{i>j}\left\{\frac{q_{i}q_{j}e^{2}}{r_{ij}}+4\epsilon_{ij}\left[{\left(\frac{\sigma_{ij}}{r_{ij}}\right)}^{12}-{\left(\frac{\sigma_{ij}}{r_{ij}}\right)}^{6}\right]\right\},$$

where $k_{x}$ denotes the force constant for each energy term, *d*, θ, ϕ represent the bond length, angle, and torsional angle within a molecule with the equilibrium bond length $d_{0}$ and angle $\theta_{0}$. In $E_{nonbond}$, *q* is the atomic partial charge, σ represents vdW radius, and ϵ denotes the vdW well-depth. Here, the PEO polymer and the lithium-ion was described using original OPLS parameters, except for the partial charges. The bis(trifluoromethane)sulfonimide (TFSI${}^{-}$) anion was modeled by recently developed OPLS parameters for ionic liquid [20,23].

**Figure 1:**
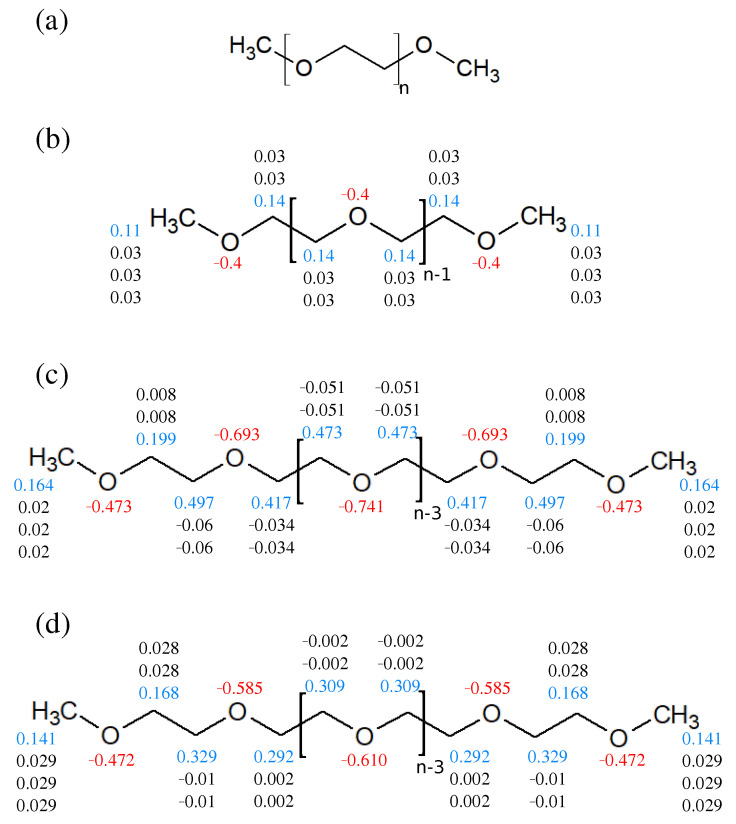
(**a**) The PEO dimethyl ether molecule as representative PEO used in this work, where n (degree of polymerization) was 10 or 20. The atomic partial charges (unit: e-) for PEO with (**b**) default OPLS, (**c**) Barbosa model, and (**d**) reparametrized OPLS^R^ models. The blue and black numbers denote the partial charges for carbon and hydrogen atoms, respectively, in the CH_2_ or CH_3_ groups.

Atomic partial charges are crucial for modeling electrolyte systems, and the restrained electrostatic potential (RESP) method has been shown to improve the predictability of non-polarizable force field in system dielectric constant calculations [37,39,40,41]. Here, we revised the OPLS force field with RESP charges, denoted as OPLS^R^, for a PEO molecule. Please note that the RESP calculation is sensitive to the molecular conformation, and thus the averaged charges are more appropriate for the model parameterization. To calculate averaged RESP charges of PEO polymer, we constructed two PEODMEs with the degree of polymerization (DP) of 10 and 20, i.e., 10 and 20 monomers, respectively, as the target PEO molecules as shown in Figure 1a. An initial configuration of a polymer melt system with 40 PEO chains in a cubic cell was established using the PACKMOL software [42]. A short MD equilibration under constant pressure and constant temperature (NPT) at 1 atm and 363 K were then performed to obtain 10 polymer chain configurations as the initial structures for RESP charge calculations of two PEO systems with DP of 10 and 20. The molecular partial charges for the two PEO systems were first calculated via density functional theory (DFT) method with B3LYP/6-311G* basis sets using Gaussian 03 package [43]. The partial atomic charges were then fitted using the RESP method via the Antechamber package [44,45]. The averaged RESP charges from 10 polymer configurations for each atom of PEO polymer were rounded to the third decimal places while maintaining neutral net charges. The RESP charges from both PEO systems in DP of 10 and 20 were compared to check the partial charge convergence with respect to the polymer length. The resulting RESP partial charges of PEO are illustrated in Figure 1 in comparison with the original OPLS model and the partial charges reported by Barbosa et al. [26].

### 2.2. Molecular Dynamics Simulation Details

The derived OPLS^R^ parameters were applied to a PEODME with DP of 64 (approximate molecular weight of 2800) as the model PEO chain to construct both pure polymer melt and solid polymer electrolyte systems. The initial configuration of the pure polymer system was constructed by randomly placing 40 PEO chains into a cubic box using the PACKMOL software [42]. The SPE system included 40 PEO chains and 160 lithium bis(trifluoromethane)sulfonimide (LiTFSI), corresponding to the O:Li^+^ ratio of 16:1. Each system was first energy minimized through the steepest descent minimization algorithm to eliminate unreasonable structures, followed by a short NVT (constant volume, constant temperature) simulation at 298K to further eliminate any high-energy configurations. The system was further heated and equilibrated at 898 K and 1 bar under NPT ensemble to facilitate the equilibration of the polymer melt. The system was then slowly annealed to 298 K with the rate of 0.08 K/ps, followed by a production run of 400 ns at 298 K and 1 bar with system configurations saved every 5 ps for further analyses. All the polymer melt and polymer electrolyte systems studied in this work are listed in Table S1–S3. All simulations were carried out using the GROMACS 5.0.4 package with an integration time step of 1 fs [46,47]. Periodic boundary conditions (PBC) were applied to the system cell in all three dimensions. The van der Waals (vdW) and short-range electrostatic potentials were cut off at 1.2 nm where the vdW interactions were smoothly shifted to zero starting from 0.8 nm. Long-range electrostatic interactions were evaluated by the Particle Mesh Ewald (PME) algorithm [48,49]. Temperature and pressure were controlled using the Nose-Hoover thermostat algorithm and Parrinello–Rahman barostats with the coupling time constants of 0.5 ps and 1 ps, respectively [50,51,52,53]. System configurations were visualized with visual molecular dynamics (VMD) software [54]. All the analyses described in the subsequent sections were performed using built-in modules of GROMACS and in-house analysis scripts.

## 3. Results and Discussion

The OPLS model revised with RESP charges, OPLS^R^, for PEO was derived as described in the Methods section. The resulting atomic partial charges of OPLS, Barbosa [26], and OPLS^R^ model for PEO are shown and compared in Figure 1. For the OPLS^R^ model, the RESP charges converge after the third ethylene oxide monomer, similar to the model derived by Barbosa [26]. Compared with the original OPLS, the partial charges of oxygen atoms are more negative in the Barbosa and OPLS^R^ resulting in larger local dipole moments, whereas the Barbosa model gives the most negative oxides among the three models. With these three force fields, the properties of PEO polymer melt systems including density, melting point (T_m_), thermal expansion coefficient, and dielectric constant were analyzed and compared to evaluate the accuracy and the reliability of the proposed OPLS^R^ model. A PEO-based SPE system was further simulated to evaluate the applicability of OPLS^R^. SPE structures, Li^+^ diffusion coefficient, and diffusion activation energy were compared among the three tested force fields and validated against experimental data.

### 3.1. Polymer Melts System

**Figure 2:**
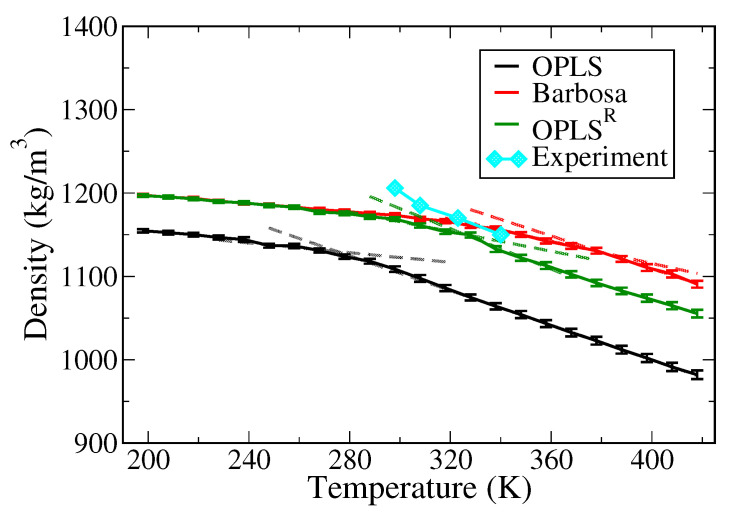
Comparison of experimental (cyan diamonds, data from [55]) and computed densities of pure PEO system as functions of temperature. The black data is the results of the OPLS model; the red and green curves correspond to the Barbosa and OPLS^R^ models, respectively. Dashed lines are fitted lines in high- and low-temperature ranges for determining the transition temperature as shown later in Figure 3.

The physical properties of PEO melt systems containing 40 PEO chains with DP of 64 were analyzed from the simulations using the three tested force fields. Figure 2 shows the density of PEO melts over a wide temperature range of 198–448 K. The resulting densities at 298 K are 1100 kg/m^3^ for the OPLS model, 1176 kg/m^3^ for the Barbosa model, and 1170 kg/m^3^ for the OPLS^R^ model. The results derived from the RESP and Barbosa models were in good agreement with the experimental value of 1200 kg/m^3^ [55], whereas the original OPLS force field underestimates by 8%. The default OPLS model exhibits a relatively small local dipole moment; thus, the calculated density deviates from the experimental value. In contrast, due to the larger dipole moments, the inter-chain interactions for the Barbosa and OPLS^R^ models are enhanced, leading to improved density values of PEO melts.

**Figure 3:**
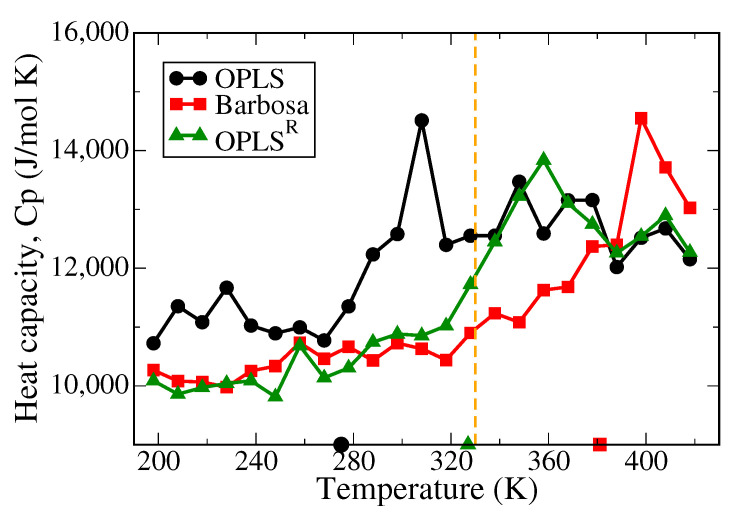
Heat capacity profiles of pure PEO systems in three different force fields as functions of temperature. The profile with black circles is the result of the OPLS model, and the ones with red squares and green triangles are the results of the Barbosa and the OPLS^R^ models, respectively. The orange dashed line denotes the experimental Tm value from [55].

Figure 3 displays the calculated specific heat capacities (C_p_) per mole of PEO chains at temperatures ranging from 198K to 418K of PEO melt systems modeled with the three tested force fields. The C_p_ was evaluated from the fluctuations of the system enthalpy *H* [56]:

(4) $$C_{p}=\frac{\langle H^{2}\rangle -{\langle H\rangle }^{2}}{k_{B}T^{2}}$$

where $k_{B}$ denotes the Boltzmann constant and *T* is the simulation temperature. The distinct peaks observed in C_p_-T profiles indicate the phase transition of the polymer melts. Moreover, the onset temperature of C_p_ rising can be related to the transition temperature determined from the density–temperature profile as shown in Figure 2. The experimental melting temperature (T_m_) of PEO is ∼330 K [55]. The results shown in Figure 3 suggest that the three tested models estimate Tm of PEO differently, with 300 K for OPLS, 400 K for the Barbosa model, and 360 K for OPLS^R^. It is noteworthy that the T_m_ overestimation for the Barbosa model is particularly severe with the relative error of around 21% of the experimental value. This is because the charges of oxygen and carbon atoms in the Barbosa model were too large compared to the other two models, leading to a greater local polarity of the PEO polymer and further resulted in an erroneous T_m_ despite the improvement in the PEO density. In contrast, the T_m_ calculated by the OPLS and the derived RESP models are more consistent with the experimental value with relative errors of 9%. For OPLS^R^, the greater dipoles also lead to slightly higher T_m_ than the experimental value; whereas the OPLS model results in a lower T_m_ due to a smaller local dipole. Yet, the onset temperature of C_p_ raising of OPLS^R^ is closer to the experimental T_m_ in comparison to that of the original OPLS model, suggesting that OPLS^R^ can describe similar phase behaviors of PEO compared to default OPLS.

**Figure 4:**
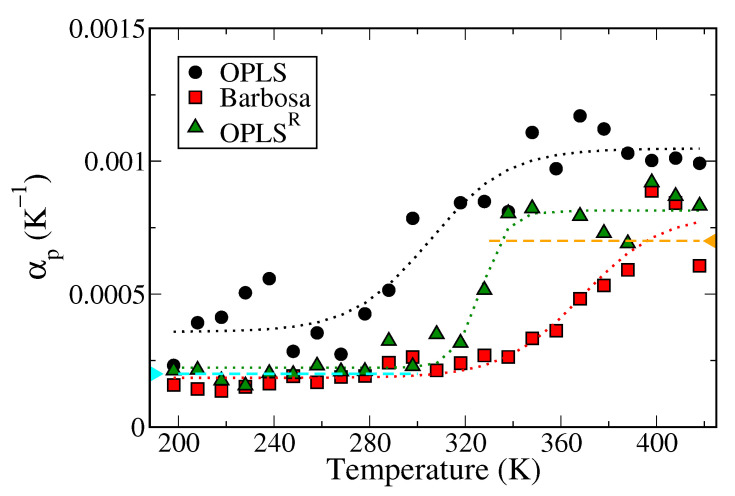
Thermal expansion coefficient of pure PEO system versus temperature. The black circles are the results of the OPLS model, the red squares and green triangles correspond to the Barbosa and the OPLS^R^ models, respectively. The cyan and orange dashed lines represent the experimental results in low and high-temperature regions, respectively. The dotted lines are the guiding sigmoidal curves fitted with logistic functions.

The thermal expansion coefficients ($\alpha_{P}$) of each PEO model were analyzed to confirm the force field validity. The thermal expansion coefficient was evaluated as [56]:

(5) $$\alpha_{P}=\frac{\left[\langle V\cdot U\rangle -\langle V\rangle \langle U\rangle +P^{2}\left(\langle V^{2}\rangle -{\langle V\rangle }^{2}\right)\right]}{k_{B}T\langle V\rangle }$$

where *U* denotes the system potential energy, *P*, *V* and *T* are the system pressure, volume and temperature, respectively, and $k_{B}$ is the Boltzmann constant. Figure 4 displays the thermal expansion coefficients ($\alpha_{P}$) as functions of temperature obtained from three different PEO models. The averaged $\alpha_{P}$ values in the high-temperature region above T_m_ and the low-temperature region below T_m_ are summarized in Table 1. In the low-temperature region of 210 K to 300 K, the expansion coefficient results of PEO in the Barbosa and OPLS^R^ model are in excellent agreement with the experimental value [57], whereas the values obtained from OPLS are marginally overestimated. In the high-temperature range, however, the results from the Barbosa model deviate more from the experimental value, where the $\alpha_{P}$ values of the Barbosa model at 400 K is already higher than the experimental data. This suggests that the Barbosa model overestimates the $\alpha_{P}$ of molten PEO above T_m_. The absence of a high-temperature plateau region in the $\alpha_{P}$-T profile of the Barbosa model can be related to the severe overestimation of T_m_ as shown in Figure 3. Surprisingly, despite that OPLS^R^ gives a slightly higher T_m_ than original OPLS model, the $\alpha_{P}$ of molten PEO above T_m_ from OPLS^R^ is closer to the experimental value than that from the original OPLS force field. Hence, the overall $\alpha_{P}$ results of the OPLS^R^ model are the most consistent with the experimental data.

The main objective of re-parameterizing atomic charges of the OPLS model for PEO is to improve the validity of polymer electrolyte simulations in which the dielectric response properties are critical. Before applying OPLS^R^ for SPE systems, we first analyzed the dielectric constants of pure PEO melts in three tested models and compared the results with experimental data [58]. The static dielectric constant ϵ is related to the fluctuations of the system dipole moment *M* and can be calculated as [59]:

(6) $$\epsilon =1+\frac{4\pi }{3}\frac{\langle M^{2}\rangle -{\langle M\rangle }^{2}}{Vk_{B}T}$$

where $k_{B}$ denotes the Boltzmann constant, *V* is the system volume, and *T* is the simulation temperature. The system dipole moment *M* was evaluated as:

(7) $$M=\sum q_{i}r_{i},$$

where the $q_{i}$ and $r_{i}$ denoted the charge and position of the *i*th atom, respectively, and the summation summed over all the particles within the system. Here, the dielectric constant ϵ was calculated using the gmxdipoles analysis tools within the GROMACS package [46,47].

The resulting dielectric constants from three different PEO models at 298 K compared with the experimental value are listed in Table 1 [58]. As reported in the literature [39], the OPLS model gives ϵ of 4.36 for pure PEO, which is severely underestimated compared to the experimental data. This indicates a poor description of the dielectric response of PEO in the original OPLS force field. The Barbosa and the newly derived OPLS^R^ models predict higher dielectric constant values of ϵ = 8.08 and 9.94, respectively, that are consistent with the experimental ϵ of 9∼11. The higher dielectric responses of the two models can be attributed to the larger local dipoles as shown in Figure 1. Changes in polymer conformations can lead to larger fluctuations for more pronounced local polarization, resulting in a greater static dielectric constant value. Combined analyses suggest that OPLS^R^ with RESP partial charges improves the predictions of bulk PEO density, thermal expansion coefficient, and dielectric response, while maintaining a reasonable description of thermotropic phase behavior.

### 3.2. Polymer Electrolyte

According to the analyses of pure PEO melt systems, although the dielectric constant and density at 298 K resulting from the Barbosa model are consistent with the experimental data, its applicability is poor at higher temperature and deviates far from the experimental values. Therefore, for the polymer electrolyte systems, we focused mainly on the comparison of OPLS and OPLS^R^ models with reported experimental data.

**Figure 5:**
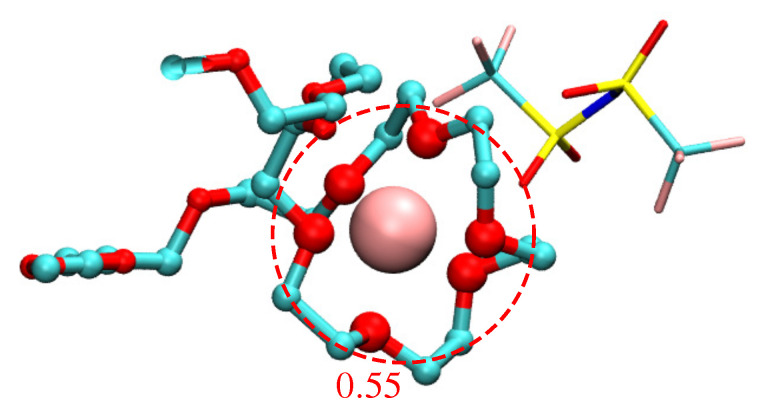
The schematics of 1 LiTFSI molecule and 1 PEO polymer chain (DP = 16). PEO polymer is illustrated using ball-and-stick representation, TFSI${}^{-}$ molecule is presented via stick model, and the pink sphere represents a lithium ion. The Li^+^ coordinating oxygen atoms of PEO are emphasized with larger size for their partial charges reduced by 0.55. The atom color codes are: oxygen in red, carbon in turquoise, nitrogen in blue, sulfur in yellow and fluoride in fluorine.

For non-polarizable force fields such as OPLS, the dynamics of electrolyte systems are known to deviate from experimental data [31,32], mainly due to the incapability of describing charge transfer between ions and polymer. A common approach to overcome this issue is through rescaling the atomic partial charges of the system by a factor of 0.5–0.8 [23,24,33,34]. Here, to examine the effects of charge transfer on partial charges in PEO/LiTFSI SPE system and to find the appropriate scaling of charges, we performed RESP calculations on a system containing one LiTFSI and one PEO chain of DP = 16, corresponding to a SPE system of O:Li^+^ = 16:1, in 10 different configurations. As demonstrated in Figure 5, PEO can wrap around Li^+^ to form a stable coordination complex. The resulting charge for Li^+^ is around +0.55 and the net charge for TFSI${}^{-}$ is ∼−0.55. Interestingly, the partial charges for oxygen atoms coordinating with Li^+^ and their nearby alkyl chains also have their charges reduced by a factor of 0.55 compared to the pure polymer system. For the PEO segment away from ions, the partial charges remain consistent with pure PEO. Therefore, we applied separate charge-scaling factors for ions and polymers in the PEO/LiTFSI SPE system. For Li^+^ and TFSI${}^{-}$, we set the scaling factor $f_{ion}=0.55$ according to the RESP calculation. Please note that in the PEO-based SPE system, there are on average 5–6 oxygen atoms coordinating with one Li^+^. Hence, for the PEO/LiTFSI SPE system with O:Li^+^ =16:1, the charge-scaling factor for PEO polymers was estimated as $f_{poly}=1/16\left[0.55\times 6+\left(16-6\right)\right]\;\approx 0.8$ to account for the averaged charge transfer effects induced by Li^+^ coordination. The resulting $f_{poly}=0.8$ is also close to the scaling factor reported in the literature [23,24].

**Figure 6:**
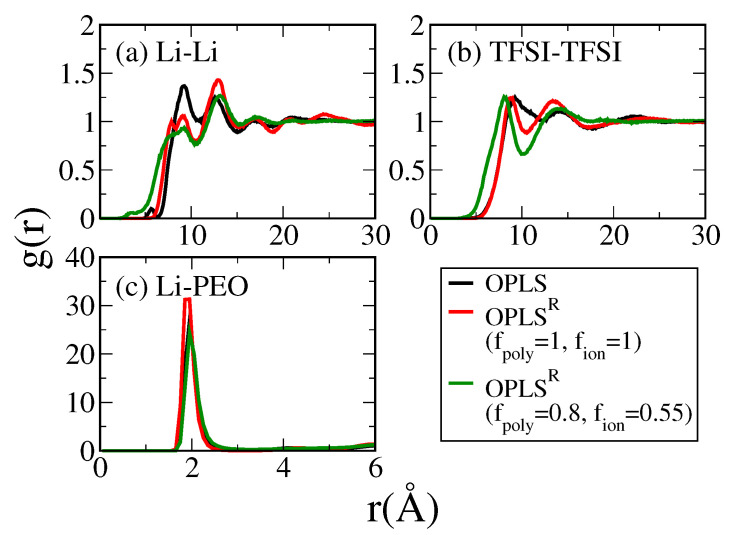
The radial distribution functions between pairs of (**a**) Li^+^-Li^+^, (**b**) TFSI${}^{-}$-TFSI${}^{-}$ and (**c**) Li^+^-PEO oxygen for PEO/LiTFSI system at 333 K in different force fields.

To validate the proposed charge-scaling scheme, we conducted equilibrium MD simulations of PEO/LiTFSI SPE systems using original OPLS, OPLS^R^ with full charge ($f_{poly}=1$, $f_{ion}=1$), and charge-rescaled OPLS^R^ ($f_{poly}=0.8$, $f_{ion}=0.55$). The SPE structures were characterized with radial distribution functions (RDF) between ions and polymers as illustrated in Figure 6. For the full-charged and charge-rescaled OPLS^R^ models, compared with the original OPLS model, the RDFs between Li^+^ exhibit attenuated first peaks at around 10 Å; whereas the RDFs between PEO and Li^+^ show enhanced peaks at 2 Å, corresponding to the first coordination shell. This indicates that the greater dipole within the OPLS^R^ model allows better solvation of Li^+^. The numbers of PEO oxygen within the first coordination shell of Li^+^, i.e., the coordination numbers CN, were 5.6 for OPLS, 5.64 for OPLS^R^, and 4.91 for charge-rescaled OPLS^R^. The resulting CN for charge-rescaled OPLS^R^ is more consistent with the values of CN ≈5 reported by polarizable MD force field and the experimental data [60,61], where the ones by OPLS and OPLS^R^ are slightly overestimated. Due to the scaled charges, the repulsion between same ions is reduced. Therefore, the first RDF peaks of Li^+^-Li^+^ and TFSI${}^{-}$-TFSI${}^{-}$ pairs slightly shift to smaller separation distance. However, comparing the full-charged and charge-rescaled OPLS^R^ models, the RDF profiles for all tested pairs are similar, suggesting that the charge-scaling has minimal effects on the SPE structural description.

The main purpose of charge-rescaling of SPE system is to improve the description of the ionic dynamics in MD simulations. To calculate the self-diffusion coefficient of Li^+^, $D_{Li^{+}}$, one of the most common approaches is through the mean square displacements (MSD, $\langle \Delta r{\left(t\right)}^{2}\rangle$) of Li^+^ via the Einstein relation [56]:

(8) $$\begin{array}{c} D=\lim_{t\to \infty }\left\{\frac{1}{6t}\langle \Delta r{\left(t\right)}^{2}\rangle \right\}, \end{array}$$

where *t* denotes the diffusion time, 〈r(t)〉 is the displacement of Li^+^ over the time span *t*, and the angle brackets represent the ensemble average. A larger $D_{Li^{+}}$ indicates better Li^+^ mobility.

**Figure 7:**
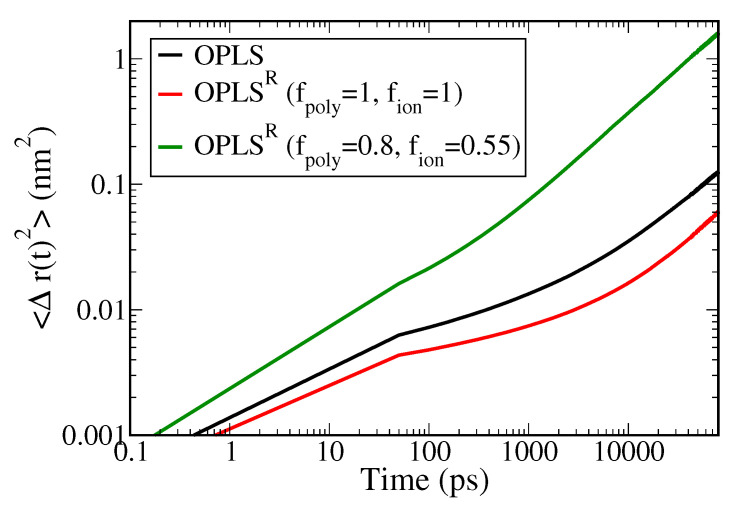
The mean square displacement profiles of Li^+^ in PEO/LiTFSI SPE system at 333 K for three tested force fields.

The resulting MSD profiles of PEO/LiTFSI SPE systems at 333 K in the default OPLS, OPLS^R^ with full charge ($f_{poly}=1$, $f_{ion}=1$), and charge-rescaled OPLS^R^ ($f_{poly}=0.8$, $f_{ion}=0.55$) force fields are shown in Figure 7 (the corresponding MSD profiles for anions are shown in Figure S1). Due to the increased dipole, the ion–polymer interactions in the OPLS^R^ model are enhanced. This further limits the ion mobility, resulting in a lower MSD profile of the full-charge OPLS^R^ model compared with the original OPLS force field. In contrast, the MSD profile for the charge-rescaled OPLS^R^ model shows an order of magnitude enhancement over the full-charged model. The calculated $D_{Li^{+}}$ values for all tested systems at 363 K, 333 K, and 298 K are listed in Table 2 in comparison to experimental data. Charge-rescaled OPLS^R^ gives $D_{Li^{+}}=2.40\times 10^{-8}$ that is most consistent with the experimental value of $3.68\times 10^{-8}$ at 333 K, whereas the original OPLS and full-charged OPLS^R^ underestimate $D_{Li^{+}}$ by one order of magnitude.

Experimentally, the contribution of Li^+^ mobility to the overall ionic conductivity is characterized by the lithium transference number (*t*${}_{Li^{+}}$). In MD simulation, *t*${}_{Li^{+}}$ can be evaluated from the diffusion coefficients of Li^+^ and counter ions as in NMR measurements [62]:

(9) $$\begin{array}{c} t_{Li^{+}}=\frac{D_{{\mathrm{Li}}^{+}}}{D_{{\mathrm{Li}}^{+}}+\;D_{{\mathrm{TFSI}}^{-}}} \end{array}$$

where $D_{{\mathrm{Li}}^{+}}$ and $D_{{\mathrm{TFSI}}^{-}}$ denote self-diffusion coefficients of the Li^+^ and TFSI${}^{-}$, respectively, evaluated from the MSD profiles shown in Figure S2–S7. As summarized in Table 2, the original OPLS and full-charged OPLS^R^ give much higher *t*${}_{Li}$ of 0.41 at 333 K than experimental data, suggesting poor descriptions of the dynamics for both Li^+^ and TFSI${}^{-}$ in the two force fields. In contrast, charge-rescaled OPLS^R^ predicts *t*${}_{Li^{+}}$ of 0.215, consistent with the experimental value of 0.24. In the charge-rescaled OPLS^R^ model, the Coulomb interactions between ions are reduced, leading to larger diffusion coefficients for both cations and anions. However, Li^+^ was still coordinated with the oxygens of the polymer chain with a larger dipole in OPLS^R^. Thus, the magnitude of the increase in D${}_{Li^{+}}$ is not as great as that in D${}_{TFSI^{-}}$. Therefore, the resulting *t*${}_{Li^{+}}$ is reduced and is more in line with the experiment.

The activation energy of Li^+^ diffusion ($E_{a}$) can be obtained from the diffusion coefficients at various temperatures according to the Arrhenius equation:

(10) $$D_{Li^{+}}\left(T\right)=D_{0}exp\left(\frac{-E_{a}}{RT}\right),$$

which is usually re-written as:

(11) $$\ln D_{Li^{+}}\left(T\right)=\ln D_{0}-\frac{E_{a}}{RT},$$

**Figure 8:**
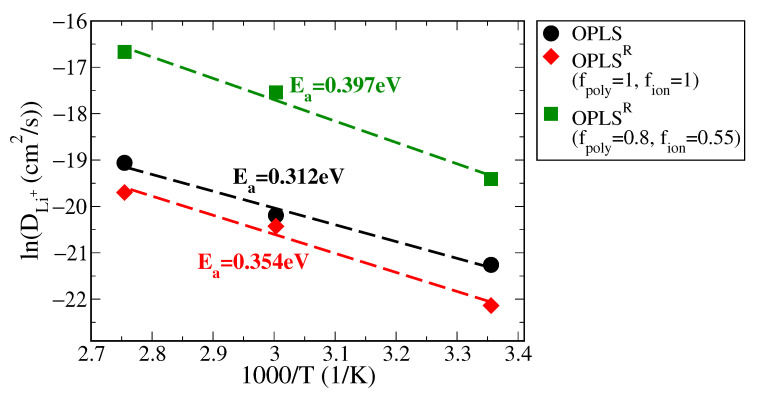
Arrhenius plot of D${}_{Li^{+}}$ versus inverted temperature for PEO/LiTFSI SPE systems in OPLS, full-charged OPLS^R^ and charge-rescaled OPLS^R^ force fields, along with the corresponding Li^+^ diffusion activation energy $E_{a}$ values.

By plotting $\ln D_{Li^{+}}\left(T\right)$ versus 1/T, the diffusion activation energy $E_{a}$ can be estimated by the slope of the linear fitted line. The resulting Arrhenius plots and the $E_{a}$ values for the PEO/LiTFSI SPE systems in the OPLS, full-charged OPLS^R^, and charge-rescaled OPLS^R^ force fields are shown in Figure 8. The calculated $E_{a}$ values for Li^+^ diffusion are close to the experimental value of ∼0.4 eV for all tested models. This validates the dynamic descriptions in the newly proposed force field. Please note that the Li^+^ transport within PEO-based SPE mainly depends on the collective motion with polymer chains. According to the structural and thermotropic analyses of PEO melts and SPE systems, the variations in partial charges have greater influences on the interactions between polymer chains than on the Li^+^ coordination environment. Hence, the activation energy for Li^+^ diffusion is less affected by the partial charges of polymers. The overall results indicate that the proposed charge-rescaled OPLS^R^ can significantly improve the description of Li^+^ mobility and correctly balances the dynamics between cations and anions.

**Figure 9:**
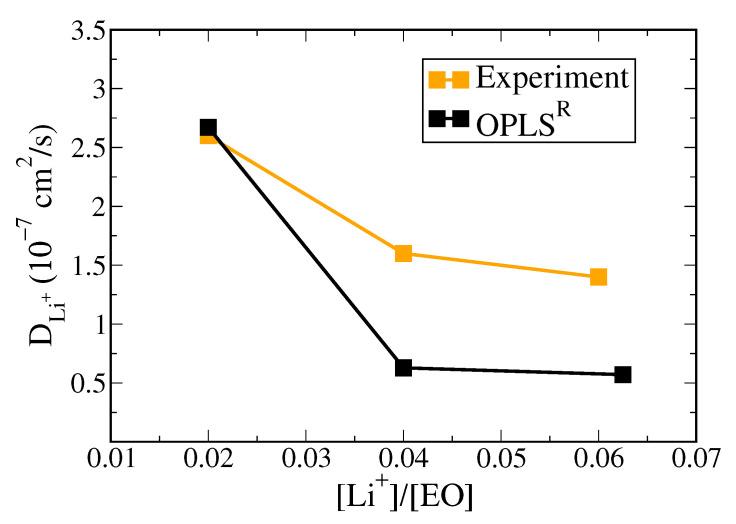
Li^+^ diffusion coefficients for PEO/LiTFSI SPE systems in charge-rescaled OPLS^R^ force field with O/Li^+^ ratio of 50, 25 and 16, corresponding to [Li^+^]/[EO] = 0.02, 0.04, and 0.0625, respectively, in comparison with experimental data from [62].

To examine the versatility of the charge-scaled OPLS^R^ model, we tested two additional PEO/LiTFSI SPE systems with various O/Li^+^ ratio of 50 and 25 at 363 K. As shown in Figure 9, all the resulting D${}_{Li^{+}}$ values (estimated from the MSD profiles for Li^+^ shown in Figure S8) are in the same order compared with the experimental data [62]. Additionally, the charge-scaled OPLS^R^ model shows similar effects of salt concentration on the Li^+^ mobility where D${}_{Li^{+}}$ decreases with increasing lithium salt content and converges after O/Li^+^ = 25. These results demonstrate the applicability of the proposed model on the PEO/LiTFSI SPE systems with a wide O/Li^+^ range.

## 4. Conclusions

Revised OPLS parameters with RESP partial charges, OPLS^R^, were proposed for long-chain PEO polymers. Series of MD simulations were conducted to validate the OPLS^R^ force field in predicting PEO melt properties compared with original OPLS model, the model developed by Barbosa et al. [26], and the experimental data. The re-parameterized RESP charge model gives a larger local dipole within PEO chains, which improves the predicted PEO density of 1170 kg/m^3^ in OPLS^R^ compared with the experimental value of 1200 kg/m^3^. Additionally, OPLS^R^ gives thermal expansion coefficients $\alpha_{P}$ more consistent with experimental values both above and below T_m_ than both the original OPLS and Barbosa models. Furthermore, the static dielectric constant of pure PEO obtained from the OPLS^R^ force field (ϵ=9.94), compared with the experimental value of 9–11, shows a significant improvement over the default OPLS model (ϵ=4.36). Combined results indicate that OPLS^R^ with RESP partial charges improves the validity of bulk PEO melt properties while retaining reasonable thermotropic phase behaviors.

The RESP revision of OPLS was further applied to MD simulations of PEO/LiTFSI polymer electrolyte systems with O:Li^+^ ratio of 16:1. Based on the RESP results, we derived separate charge-scaling factors for ions and polymers ($f_{poly}=0.8$, $f_{ion}=0.55$) in the PEO/LiTFSI SPE system. According to RDF analyses, applying the charge-scaling factors has limited effects on the Li^+^ coordination structures within SPE systems. However, due to the reduced Coulomb interaction, the ion mobilities are greatly enhanced. The diffusion coefficient $D_{Li^{+}}$ of $2.40\times 10^{-8}$ at 333 K from charge-rescaled OPLS^R^ is the most consistent with the experimental value of $3.68\times 10^{-7}$ among the tested PEO models. The derived lithium transference number and the activation energy of Li^+^ diffusion in the OPLS^R^ force field are also in good agreement with the reported experimental data. The OPLS^R^ model is also applicable for PEO/LiTFSI SPE systems with a wide O/Li^+^ range of 16 to 50. These results demonstrate the versatility and the validity of the proposed charge-rescaled OPLS^R^ for the description of Li^+^ mobility and balances between cation and anion dynamics within SPE systems. The proposed parameterization and the charge-scaling rules can also benefit future simulation studies on polymer electrolyte systems using non-polarizable force fields.

## Figures and Tables

**Table 1:** Thermal expansion coefficient $\alpha_{P}$ above and below T_m_ and the dielectric constant evaluated at 298 K for the PEO melt systems in the three tested force fields compared with experimental values (data from [57,58]).

	$\alpha_{P}$ (T > T_m_) (K${}^{-1}$)	$\alpha_{P}$ (T < T_m_) (K${}^{-1}$)	Dielectric Constant (F/m)
Experiment [57,58]	$7\times 10^{-4}$	$2\times 10^{-4}$	9–11
OPLS	$1.04\left(\pm 0.07\right)\times 10^{-3}$	$3.65\left(\pm 0.84\right)\times 10^{-4}$	4.36 (±0.11)
Barbosa	−	$1.85\left(\pm 0.21\right)\times 10^{-4}$	8.08 (±0.67)
OPLS^R^	$8.43\left(\pm 0.45\right)\times 10^{-4}$	$2.23\left(\pm 0.22\right)\times 10^{-4}$	9.94 (±0.56)

**Table 2:** Diffusion coefficient for Li^+^, and lithium transference number (*t*${}_{Li}$) for PEO/LiTFSI SPE system in OPLS, full-charged OPLS^R^ and charge-rescaled OPLS^R^ force fields compared with experimental data. The reported experimental values were measured for the PEO/LiTFSI SPE systems with O/Li^+^ ratio of 18 at 333 K (data from [11]) and 16.7 at 363K (data from [62]).

Unit: cm${}^{2}$ s${}^{-1}$	363 K	333 K	298 K	*t*${}_{{Li}^{+}}$ (333 K)
Experiment	$1.40\times 10^{-7}$	$3.68\times 10^{-8}$	−	0.24
OPLS	$5.27\times 10^{-9}$	$1.70\times 10^{-9}$	$5.85\times 10^{-10}$	0.41
OPLS^R^ ($f_{poly}=1.0,f_{ion}=1.0$)	$2.78\times 10^{-9}$	$1.34\times 10^{-9}$	$2.43\times 10^{-10}$	0.363
OPLS^R^ ($f_{poly}=0.8,f_{ion}=0.55$)	$5.76\times 10^{-8}$	$2.40\times 10^{-8}$	$3.73\times 10^{-9}$	0.215

## Data Availability

The study did not report any data.

## Supplementary Materials

The following are available online at https://www.mdpi.com/article/10.3390/polym13071131/s1, Figure S1: The mean square displacement profiles of TFSI in PEO/LiTFSI SPE system at 333 K for three tested force fields, Figure S2: The mean square displacement profiles of Li^+^ in PEO/LiTFSI SPE system at different temperature of default OPLS force field, Figure S3: The mean square displacement profiles of Li^+^ in PEO/LiTFSI SPE system at different temperature of OPLS^R^ force field with scaling factor ($f_{poly}=1$, $f_{ion}=1$), Figure S4: The mean square displacement profiles of Li^+^ in PEO/LiTFSI SPE system at different temperature of OPLS^R^ force field with scaling factor ($f_{poly}=0.8$, $f_{ion}=0.55$), Figure S5: The mean square displacement profiles of TFSI in PEO/LiTFSI SPE system at different temperature of default OPLS force field, Figure S6: The mean square displacement profiles of TFSI in PEO/LiTFSI SPE system at different temperature of OPLS^R^ force field with scaling factor ($f_{poly}=1$, $f_{ion}=1$), Figure S7: The mean square displacement profiles of TFSI in PEO/LiTFSI SPE system at different temperature of OPLS^R^ force field with scaling factor ($f_{poly}=0.8$, $f_{ion}=0.55$), Figure S8: The mean square displacement profiles of Li+ in PEO/LiTFSI SPE system at [EO]/[Li^+^] ratio of OPLS^R^ force field with scaling factor ($f_{poly}=0.8$, $f_{ion}=0.55$), Table S1: Pure PEO systems, Table S2: Electrolyte system ([EO]/[Li^+^] =16), Table S3: Electrolyte system (OPLS^R^ force field with different [EO]/[Li^+^] ratio at 363 K).

## Abbreviations

The following abbreviations are used in this manuscript:

MD	molecular dynamics
OPLS	optimized potentials for liquid simulations
RESP	the restrained electrostatic potential
OPLS^R^	OPLS model with RESP charges
PEO	polyethylene oxide
PEODME	poly(ethylene oxide) dimethyl ether
DP	degree of polymerization
SPE	solid polymer electrolyte
