# Supplementary material for: Revised Atomic Charges for OPLS Force Field Model of Poly(Ethylene Oxide): Benchmarks and Applications in Polymer Electrolyte
Source: Polymers (Basel). 2021 Apr 2;13(7):1131. doi: 10.3390/polym13071131 (PMC8037826; doi:10.3390/polym13071131)
Supplement: Supplementary file 1 [file polymers-13-01131-s001.pdf]

Supplementary Material

Revised Atomic Charges for OPLS Force Field Model of Poly(Ethylene Oxide): Benchmarks and Applications in Polymer Electrolyte

Chan-En Fang [1,†], Yi-Chen Tsai [1,2,†], Christoph Scheurer [3,†] and Chi-cheng Chiu [1,2,†,*]

1 Deparment of Chemical Engineering, National Cheng Kung University, Tainan 70101, Taiwan; samuel083826@gmail.com(C.-E. F.); a4775324@gmail.com (Y.-C. T.)

2 Hierarchical Green-Energy Materials (Hi-GEM) Research Center, National Cheng Kung University, Tainan 70101, Taiwan

3 Chair for Theoretical Chemistry and Catalysis Research Center, Technische Universität München, Lichtenbergstrasse 4, D-85747 Garching, Germany

* Correspondence: ccchiu2@mail.ncku.edu.tw

† These authors contributed equally to this work.

1. Simulation system list

Table S1. Pure PEO systems

	**OPLS**	**BARBOSA**	**RESP**
**Different T**	20ns for each T	20ns for each T	20ns for each T
**298K equilibrium**	150ns	400ns	700ns

Table S2. Electrolyte system ($[EO]/[Li^+]$ =16)

	**OPLS**	**RESP(1/1)**	**RESP(0.8/0.55)**
**298K**	300ns	300ns	200ns
**333K**	300ns	300ns	200ns
**363K**	300ns	300ns	500ns

Table S3. Electrolyte system ($OPLS^R$ force field with different $[EO]/[Li^+]$ ratio at 363K)

System([EO]/[Li⁺])	***Simulation time***
16 (0.8/0.55)	500ns
25 (0.8/0.55)	500ns
50 (0.8/0.55)	500ns

2. Diffusion of TFSI (333K, [EO]/[Li^+]=16)

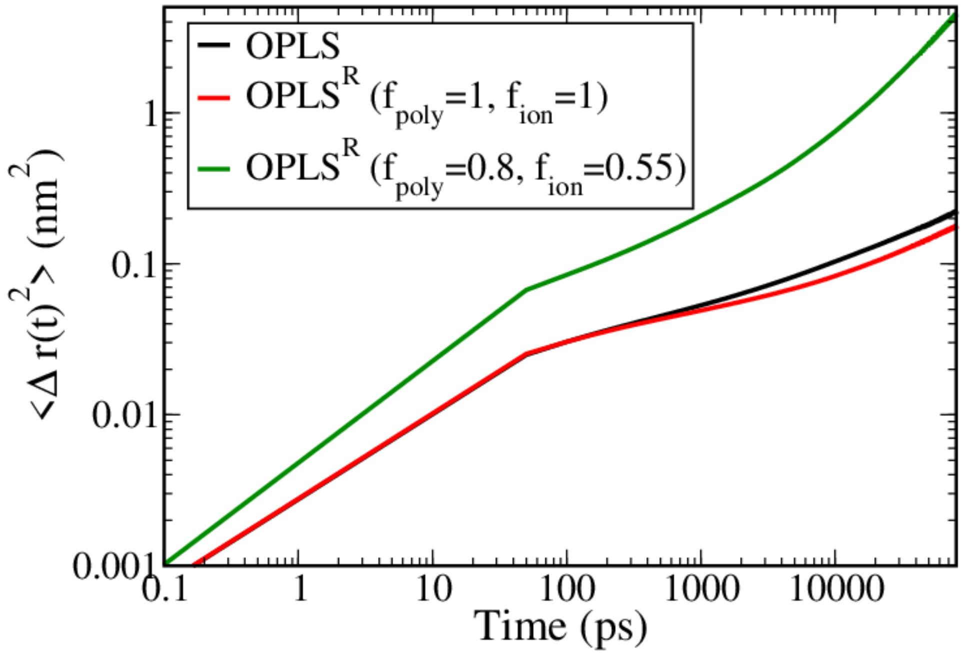

Figure s1. The mean square displacement profiles of TFSI in PEO/LiTFSI SPE system at 333K for three tested force fields.

3. Diffusion of Li^+ at different temperature ([EO]/[Li^+]=16)

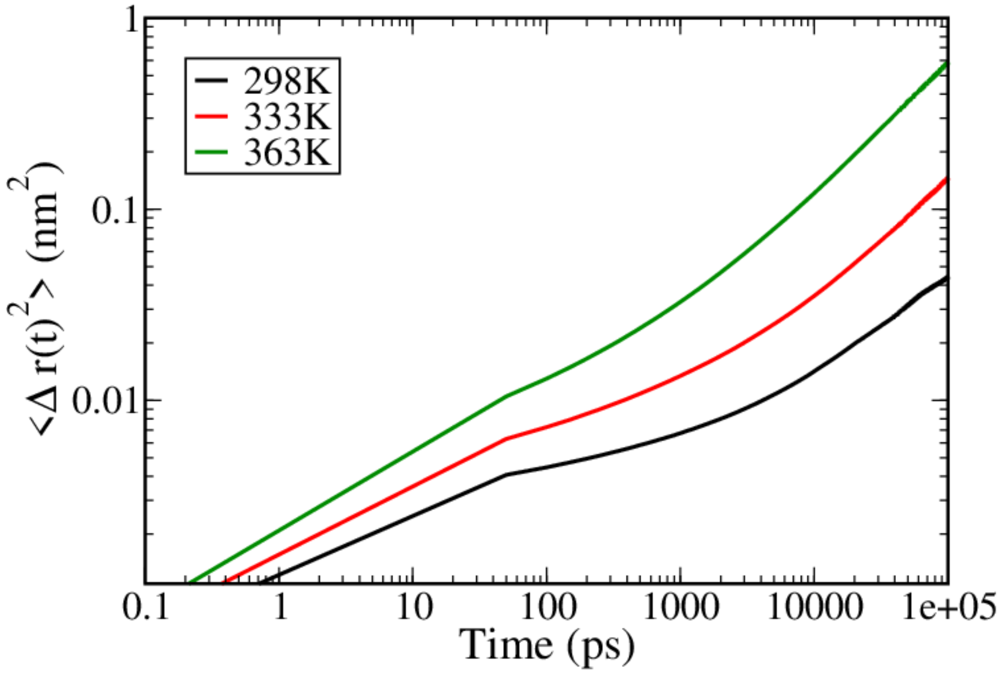

Figure s2. The mean square displacement profiles of Li^+ in PEO/LiTFSI SPE system at different temperature of default OPLS force field.

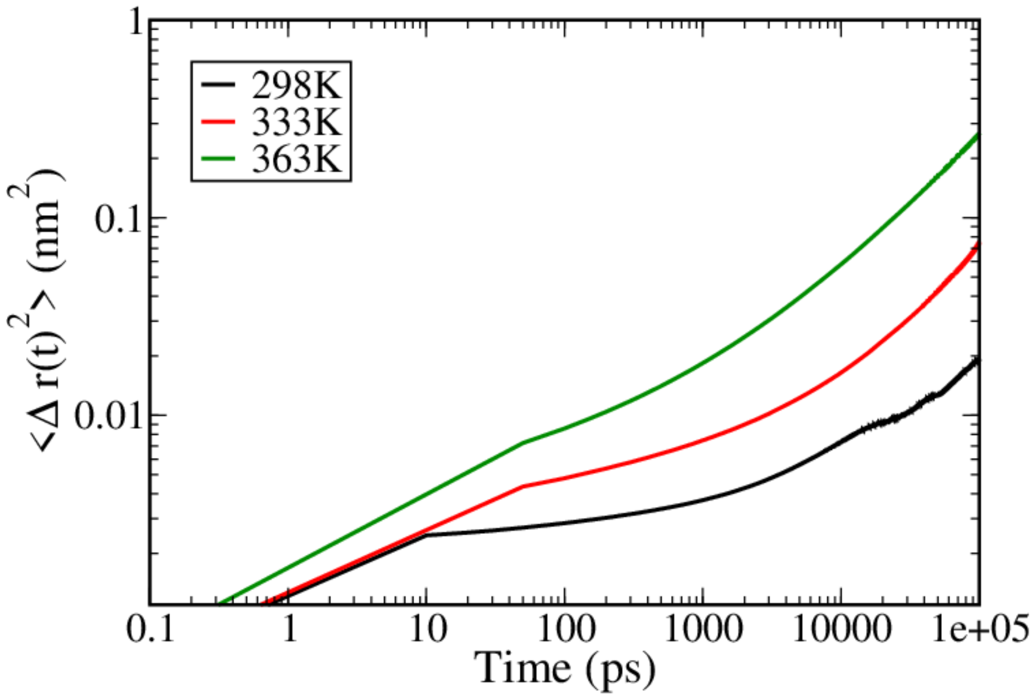

Figure s3. The mean square displacement profiles of Li^+ in PEO/LiTFSI SPE system at different temperature of $OPLS^R$ force field with scaling factor (f_{poly} = 1, f_{ion} = 1).

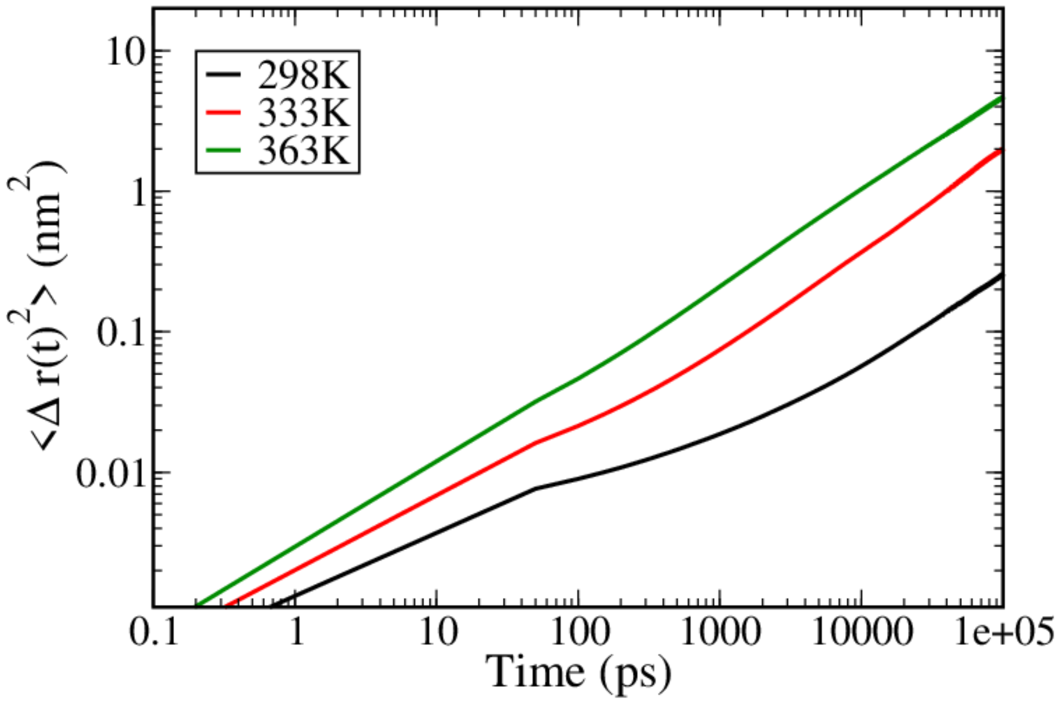

Figure s4. The mean square displacement profiles of Li^+ in PEO/LiTFSI SPE system at different temperature of $OPLS^R$ force field with scaling factor (f_{poly} = 0.8, f_{ion} = 0.55).

4. Diffusion of TFSI at different temperature ([EO]/[Li$^+$]=16)

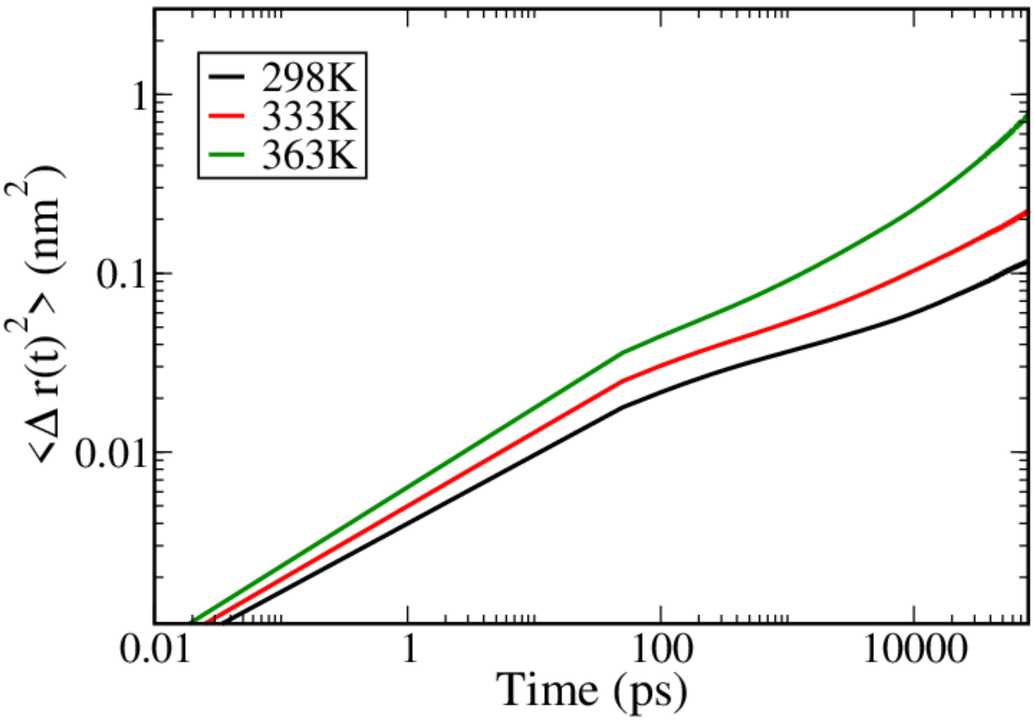

Figure s5. The mean square displacement profiles of TFSI in PEO/LiTFSI SPE system at different temperature of default OPLS force field.

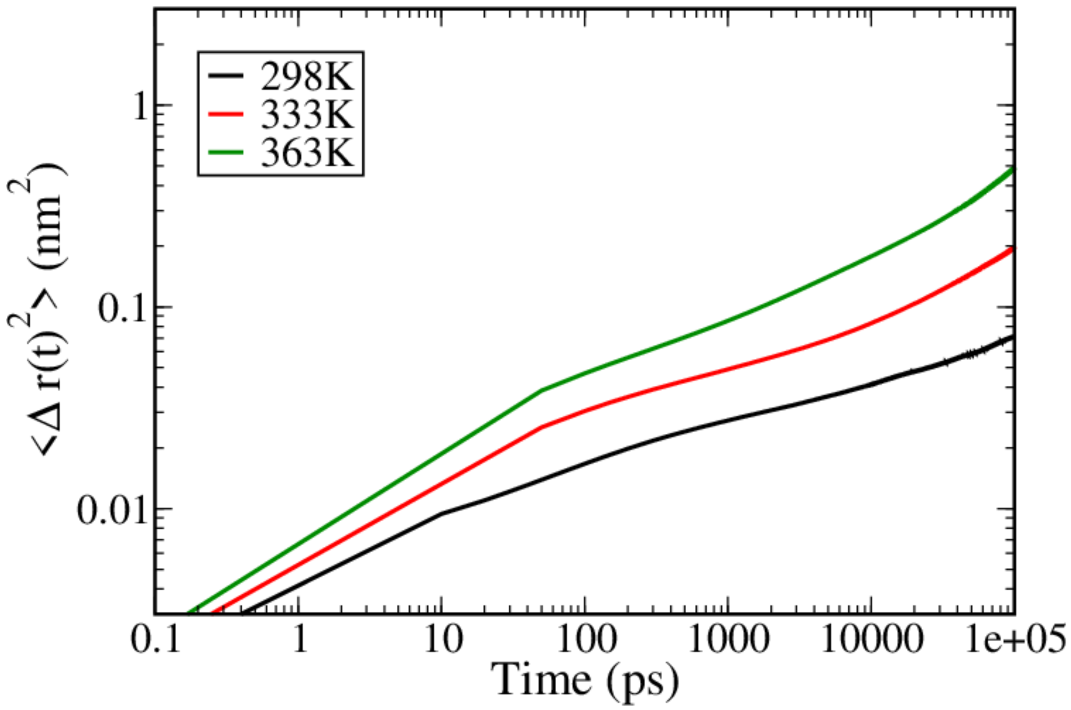

Figure s6. The mean square displacement profiles of TFSI in PEO/LiTFSI SPE system at different temperature of OPLS^R force field with scaling factor (f_{poly} = 1, f_{ion} = 1).

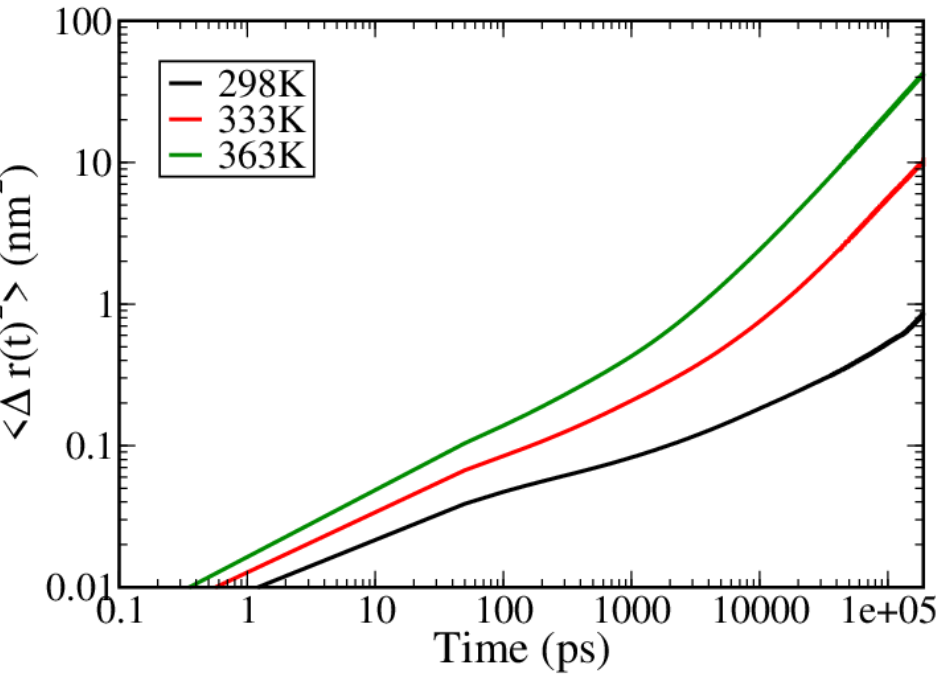

Figure s7. The mean square displacement profiles of TFSI in PEO/LiTFSI SPE system at different temperature of OPLS^R force field with scaling factor (f_{poly} = 0.8, f_{ion} = 0.55).

5. Diffusion of Li^+ in different [EO]/[Li^+] ratio at 363K

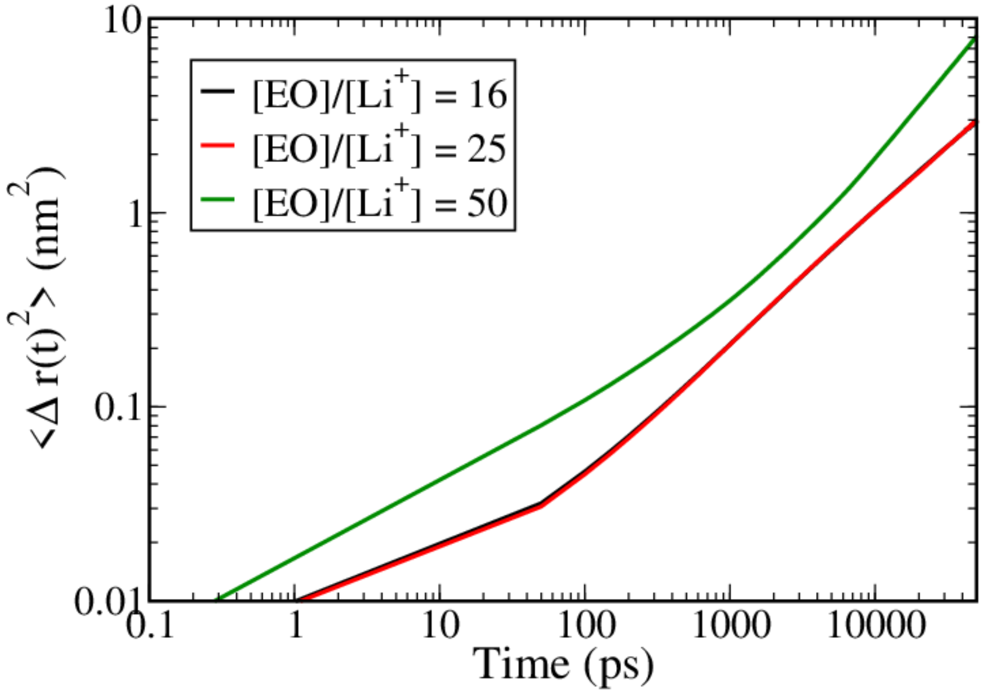

Figure s8. The mean square displacement profiles of Li^+ in PEO/LiTFSI SPE system at [EO]/[Li^+] ratio of OPLS^R force field with scaling factor (f_{poly} = 0.8, f_{ion} = 0.55).
